# The Pretreatment of Lignocelluloses With Green Solvent as Biorefinery Preprocess: A Minor Review

**DOI:** 10.3389/fpls.2021.670061

**Published:** 2021-06-08

**Authors:** Xiaoyan Yin, Linshan Wei, Xueyuan Pan, Chao Liu, Jianchun Jiang, Kui Wang

**Affiliations:** ^1^Institute of Chemical Industry of Forest Products, Chinese Academy of Forestry, Nanjing, China; ^2^National Engineering Laboratory for Biomass Chemical Utilization, Nanjing, China

**Keywords:** lignocellulose, cellulose, hemicellulose, lignin, green solvent, pretreatment

## Abstract

Converting agriculture and forestry lignocellulosic residues into high value-added liquid fuels (ethanol, butanol, etc.), chemicals (levulinic acid, furfural, etc.), and materials (aerogel, bioresin, etc.) *via* a bio-refinery process is an important way to utilize biomass energy resources. However, because of the dense and complex supermolecular structure of lignocelluloses, it is difficult for enzymes and chemical reagents to efficiently depolymerize lignocelluloses. Strikingly, the compact structure of lignocelluloses could be effectively decomposed with a proper pretreatment technology, followed by efficient separation of cellulose, hemicellulose and lignin, which improves the conversion and utilization efficiency of lignocelluloses. Based on a review of traditional pretreatment methods, this study focuses on the discussion of pretreatment process with recyclable and non-toxic/low-toxic green solvents, such as polar aprotic solvents, ionic liquids, and deep eutectic solvents, and provides an outlook of the industrial application prospects of solvent pretreatment.

## Introduction

Environmental problems due to the lack and excessive exploitation of globalized fossil energy resources have drawn more and more attention to green and renewable energy resources that can replace fossil energy (Zhang et al., 2017, 2020a). Lignocellulosic biomass is believed as one of the most promising renewable carbon resources that could be converted into liquid fuels and chemicals. Agricultural and forestry wastes, such as corn cob, bagasse, hay, straw, rice husk, wood chips, and other biomass, are abundant, low-cost, and easy to access. By the bio-refining process, they can be massively produced into bio-ethanol, butanol, and other liquid fuels, or be used to prepare high value-added chemicals and degradable materials, etc. (Cherubini, 2010; Rinaldi et al., 2016; Rastogi and Shrivastava, 2017). It is effective to promote the process of energy security, energy conservation, emission reduction, and environmental protection for economic and social benefits by carrying out bio-refinery research on lignocellulosic agricultural and forestry residues.

Lignocellulosic biomass is mainly composed of three major components, namely, 40–55% cellulose, 25–35% hemicellulose, and 15–30% lignin (Mooney et al., 1999; Jorgensen et al., 2007; Kim et al., 2016). Cellulose is the skeleton part of the biomass structure, encapsulated by lignin and hemicellulose (Hokkanen et al., 2016). In other words, lignin is the protective layer of hemicellulose and cellulose. They are connected to each other by hydrogen bonds and anisole bonds (Arantes and Saddler, 2011; Du et al., 2013), forming a lignin–carbohydrate complex structure by intermolecular forces, such as van der Waals force, which prevents enzymes and chemical agents from directly contacting cellulose (Zhao et al., 2017). In addition, owing to the complex structure of lignocellulosic biomass, the efficiency of biorefining is reduced by the inhibitory effect of depolymerization products of hemicellulose and lignin on lignocellulose hydrolysis (Yat et al., 2008; Yu et al., 2014; Yoo et al., 2017). Moreover, studies have shown that the presence of lignin has a greater effect on the anti-biodegradation characteristics of lignocellulose cell walls (Han et al., 2020). According to reports, lignin can produce unproductive binding with cellulase, causing the enzyme to adsorb cellulose ineffectively through hydrophobic interaction, electrostatic interaction, and hydrogen bonding interaction, which inhibits the biotransformation of lignocellulosic biomass (Kapoor et al., 2015; Saini et al., 2016; Li and Zheng, 2017; dos Santos et al., 2019). Therefore, an effective pretreatment process can selectively and efficiently remove hemicellulose and lignin, destroy the structure of lignocellulose, reduce the degree of cellulose polymerization, and increase the effective contact of cellulose and chemical reagents on cellulose, which makes the bio-refinery process more efficient and environmentally benign (Yang et al., 2016; Syaftika and Matsumura, 2018; Zhang et al., 2020c).

This review mainly introduces the latest research achievements of researchers in recent years on biomass delignification, including three major aspects of solvent pretreatment that commonly used common solvent (alcohol, ketone, etc.) pretreatment method, novel solvent (ionic liquid and eutectic solvent) pretreatment method, and recyclable non-toxic/low-toxic green solvent pretreatment methods. As compared, the traditional pretreatment with biological, physical, chemical and combined methods were also introduced here for better understanding of the efficient process. Notably, we significantly discuss the pretreatment methods of lignocellulosic biomass with recyclable non-toxic/low-toxic green solvents that are not carcinogenic or mutagenic to humans and laboratory animals, and degrade easily without affecting the environment (Anastas and Eghbali, 2010; Popek, 2018; Zimmerman et al., 2020), expecting to provide strategy and reference for industrial application prospects.

## Traditional Pretreatment Methods

The pretreatment process has been proved to be an extremely important part of the bio-refinery process (Zhai et al., 2019). Various pretreatment methods have been traditionally developed, mainly including biological, physical, chemical, and combination methods with the advantages and disadvantages shown in Table 1.

**Table 1:** The advantages and disadvantages of traditional pretreatment methods.

Pretreatment methods	Advantages	Disadvantages	References
Biological method	Mild treatment conditions, high efficiency of enzymatic hydrolysis	Long processing time	Chen et al., 2017b
Physical method	Increase the reaction area	Lignin cannot be removed	Aguilar-Reynosa et al., 2017
Combination method	Improve response efficiency, reduce energy consumption	Complicated operation, immature technology	Teymouri et al., 2005
Acid pretreatment	Degradation of large quantities of hemicellulose	Corrosion of the equipment, pollution of the environment	Rezania et al., 2020
Alkaline pretreatment	Degradation of large amounts of lignin	Long pretreatment time	Cheah et al., 2020
Common organic solvents	Removing large amounts of lignin and hemicellulose	High toxicity, pollution of the environment	Zhang et al., 2016c
ILs	High thermal stability, low vapor pressure	High cost, high toxicity	Khan et al., 2021
DES	High thermal stability, low cost, low toxicity	High viscosity, not easy to recycle	Satlewal et al., 2018

### Biological Method

The biological method mainly refers to the pretreatment of lignocellulose using microbial technology to destroy its structure, followed by removing lignin, which significantly improves the utilization rate of cellulose (Chen et al., 2017). Microorganisms, such as white rot fungi, brown rot fungi, soft rot fungi, or their combinations, are applied into the pretreatment process (Dias et al., 2010; Tian et al., 2018). Singh et al. pretreated wheat straw with *Phanerochaete chrysosporium*, which achieved 30% of total lignin removal rate within 3 weeks, while the total sugar content was reduced by about 27% compared with untreated wheatgrass (Singh et al., 2011b). Saha et al. (2016) pretreated corn stover with white rot fungus strain *Pycnoporus sanguineus* NRRL-FP-103506-Sp at 28°C and 74% humidity for 30 days and found that the lignin loss rate reached 51 ± 1.2%, and the hemicellulose and cellulose loss rates were 50.7 ± 2.1% and 25.4 ± 0.3%, respectively. Despite the mild and green reaction conditions of the biological method, it is still difficult to achieve large-scale industrial applications because of extreme long treatment time, low efficiency, few types of microorganisms that degrade lignin, and strict degradation conditions (Chen et al., 2017).

### Physical and Combination Methods

Physical method mainly includes mechanical comminution (Sun and Cheng, 2002), radiation pretreatment (Yang and Wang, 2018), microwave pretreatment (Liu et al., 2021), freeze pretreatment (Chang et al., 2011), etc., which significantly improves the enzymatic hydrolysis efficiency and tunes the reaction time. However, the physical method still has some disadvantages, such as high energy consumption and low lignin removal rate (Aguilar-Reynosa et al., 2017). It has been reported that the sugar yield of glucose and xylose/mannose was 59.67 and 23.82%, respectively, after 30 min of planetary ball milling of Douglas-fir forest residuals (Gu et al., 2018). Monschein adopted the steam explosion method for pretreatment of wheat straw (Monschein and Nidetzky, 2016). Inevitably, only 36% of the removal rate of lignin was achieved, accompanying with 24.1 and 38.7% of hemicelluloses and cellulose loss rates, respectively. In order to receive effective pretreatment, most of the existing physical pretreatment methods are conducted in combination with chemical or biological methods (Bals et al., 2010). Compared with wet ball milling, the enzymatic hydrolysis efficiency of the pretreatment of corn stover by the alkaline milling method was increased by 110% (Kumar and Sharma, 2017). The combination method has the characteristics of greatly destroying the structure of lignocellulose, effectively removing lignin, improving the efficiency of enzymolysis, and reducing the loss of equipment, but there are still some shortcomings that limit its industrial application, such as insufficient technology, high energy consumption, difficulty to operate, and pollution (Teymouri et al., 2005).

### Chemical Method

The chemical method is usually defined as the process of destroying lignocellulosic dense cell wall structures with chemical reagents, such as acid, alkali, and organic solvent to remove hemicellulose or lignin.

#### Acid Pretreatment

**Figure 1:**
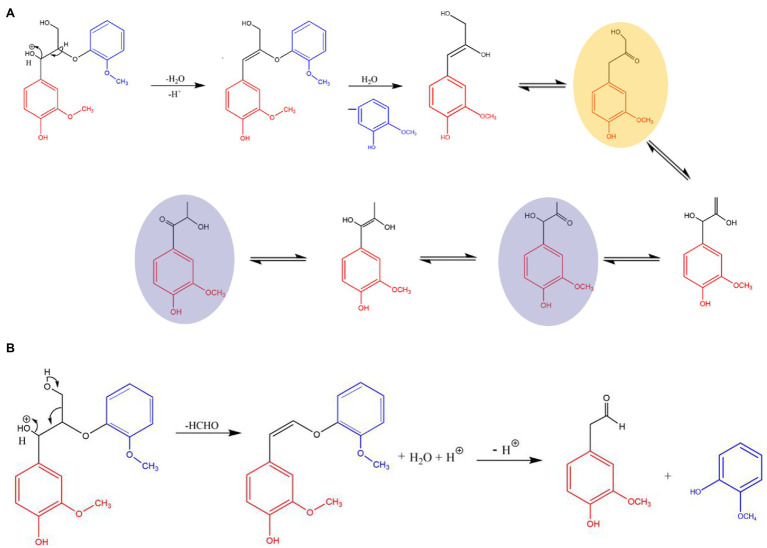
The main reaction mechanism in acid catalyzed organic solvent pretreatment. **(A)** β-O-4 linkages cleavage to form ω-guaiacylacetone (yellow) and then Hibbert’s ketones (purple). **(B)** The cleavage of β-O-4 linkages and the elimination of formaldehyde.

Acid treatment is usually conducted with dilute acid (concentration within 5%), concentrated acid (concentration above 30%), and organic acid, which could destroy the compact structure of lignocelluloses and improve the efficiency of enzymatic hydrolysis (Pedersen et al., 2011; Wettstein et al., 2012). When performing dilute and concentrated acid treatment, the treatment process may suffer higher reaction temperature (generally between 100 and 240°C; Teramoto et al., 2008; Zhu et al., 2015), high reaction pressure, or longer reaction time (2–10 h) under normal pressure (Chen et al., 2017). Notably, organic acids, such as oxalic acid and citric acid, were also commonly used in the pretreatment process (Qin et al., 2012). The mechanisms of acid hydrolysis on (i) breaking the lignin–carbohydrate complex bonds; (ii) cellulose fibrils and hemicellulose are dissolved by breaking the ordered hydrogen bonds between sugar chains; (iii) weakly hydrolyze cellulose and hemicellulose to low degree of polymerization fragments; and (iv) the acetyl group is removed from hemicellulose to form acetic acid (Zhang et al., 2007). Figure 1 illustrates the main reaction mechanism in acid catalyzed organic solvent pretreatment (Zhou et al., 2018). Teramoto et al. (2008) pretreated eucalyptus wood flour with 1% acetic acid and treated it at 200°C for 1 h. Only 12% of lignin was removed, accompanied with 67% degradation rate of hemicelluloses. Besides, the sugar conversion rate of cellulose could reach 96.6%. However, inevitable equipment corrosion and environmental pollution still restrict the industrial application of acid pretreated process.

It should be noted that high temperature liquid water is believed as a green acid solvent in the pretreatment process, during which the reaction temperature is generally conducted between 150 and 350°C with saturated vapor pressure (Lu et al., 2016). Generally, pretreatment with compressed liquid water as the reaction medium could effectively remove hemicellulose, change the structure of lignin by condensation reaction, and thus, change the non-productive binding of lignin to cellulase (Ko et al., 2015). It is has been reported that when the *Eucalyptus grandis* wood powder is pretreated at 200°C for 20 min with 5 wt% substrate concentration, more than 38.2% removal rate of lignin was achieved with up to 91.5% retention rate of cellulose (Yu et al., 2010). Despite its environmentally benign superiority, the compressed liquid water pretreatment process still suffers from high water and energy consumption and low lignin removal rate.

#### Alkaline Pretreatment

**Figure 2:**
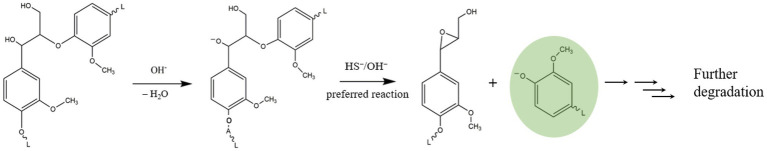
The cleavage of βaryl ether bonds under alkaline conditions.

The alkaline pretreatment method is one of the most effective delignification methods, owing to its effectiveness on breaking the anisole bond between lignin and xylan, and ability to degrade glycosides (Li et al., 2010), destroy the structure of lignin, and reduce the degree of polymerization and crystallinity of cellulose (Zhang et al., 2020b). Alkaline reagents, such as NaOH, Na_2_CO_3_, Ca(OH)_2_, KOH, and ammonia, were reported in the alkaline pretreatment process (Sun et al., 2014; de Carvalho et al., 2016; Mirmohamadsadeghi et al., 2016; Yuan et al., 2018; Júnior et al., 2020). The mechanism of alkaline hydrolysis is that the uronic ester bond that functions as cross-link is broken by saponification, and the crosslinks between the xylan chain and other polymerization units are broken. Finally, lignin is removed and cellulose and hemicellulose are retained (Tarkow and Feist, 1969). Figure 2 illustrates the main reaction mechanism under alkaline conditions (Rinaldi et al., 2016). de Carvalho et al. (2016) pretreated bagasse at 175°C for 1.5 h with a 15% NaOH solution, and more than 90% lignin removal rate was obtained. Meanwhile, the hemicellulose and cellulose removal rates were achieved at 45.3 and 11.1%, respectively. Furthermore, the yield of glucose could reach up to 51.1% by the saccharification of pretreatment liquid for 24 h. In addition, interesting studies have shown that under room temperature and normal pressure conditions, soaking corn stover in aqueous ammonia for 10–60 days could remove 55–74% of lignin without stirring, retaining 85% of the xylan and with the glucan remaining almost unchanged (Kim and Lee, 2005).

Compared with the acid pretreatment method, alkaline pretreatment requires milder conditions, is a relatively easier process, and has better treatment effect, which can remove most of the lignin and part of the hemicellulose, swell the cellulose, and improve enzymolysis rate (Sindhu et al., 2014). However, it has disadvantages such as longer treatment time and having a more complicated post process (Bali et al., 2015). Moreover, the low purity of lignin separated by alkali pretreatment significantly limits its application in actual production (Zhao et al., 2017).

#### Organic Solvent Pretreatment Methods

Traditional organic solvent pretreatment methods usually adopt alcohols, ketones, phenols, ionic liquids, eutectic solvents, and PASs as solvent.

##### Alcohol and Phenol Pretreatment Methods

Due to its outstanding effect on the breaking of hydrogen bond, ether bond and glycoside, organic solvent pretreatment can remove large amounts of lignin and almost all hemicellulose, thereby improving the efficiency of enzymatic hydrolysis. Moreover, the pretreatment effect can be further improved by adding a catalyst (Muurinen, 2000; Koo et al., 2011; Meng et al., 2020a). Generally, organic solvents adopted during pretreatment are ethanol, acetone, phenols, etc. (Zhao et al., 2009; Espinoza-Acosta et al., 2014; Vallejos et al., 2015). In recent years, ethanol has been favored by a wide range of researchers because of its low cost, low toxicity, easy recyclability, and bio-yield (Espinoza-Acosta et al., 2014; Zhou et al., 2018). *Liriodendron tulipifera* was pretreated in a 50% ethanol solvent system with dilute sulfuric acid as catalyst at 150°C for 30 min and 80.2% of lignin was removed (Jang et al., 2016). Notably, enzymolysis efficiency exceeded 80% after the pretreatment liquid was fermented. Wang et al. pretreated poplar powders with a acid-catalyzed biphasic water/phenol system under optimized conditions (98% sulfuric acid accounts for 3.5% by weight of wood, 1:8 of solid/liquid ratio w/v, 2:3 of phenol/water v/v, 120°C, and 1 h; Wang et al., 2020a). The removal rate of original lignin was about 90%, the retention rate of original cellulose was more than 96%, and the retention rate of xylose was 77%.

Despite the efficiency of organic solvent pretreatment, the process still suffers from volatility, flammability, environmental pollution, and energy consumption of organic solvents.

##### Ionic Liquid Pretreatment Method

Defined as a new type of solvent, ILs are generally composed of organic cations (imidazolium, piperidinium, ammonium, etc.) and organic or inorganic anions (Zhang et al., 2012; Khan et al., 2021), and present as liquid phase at room temperature. Due to its efficiency on selective dissolution of lignin and cellulose (Muhammad et al., 2015; Khan et al., 2016; Xu and Wang, 2020), renewability, and biodegradability, ILs are widely applied to separate the major components of lignocellulosic biomass (Kilpeläinen et al., 2007; Hou et al., 2012, 2013; Xu et al., 2018). Compared with ordinary solvents, ILs have the characteristics of being less volatile, and having lower vapor pressure, higher electrochemical stability, and higher thermal stability (Khan et al., 2021). It is worth noting that researchers can design functionalized ionic liquids by the demand of the reaction system to improve its selectivity and efficiency. Achinivu et al. reported that [Pyrr]OAc prepared with pyridine, pyrrole, N-methylimidazole, and acetic acid could extract more than 70% of lignin by treating corn stover at 90°C for 24 h, and that the recycle rates of glycan, xylan, and arabinan could reach 82.17, 65.53, and 68.95%, respectively (Achinivu et al., 2014). Despite the outstanding advantages mentioned above, ILs still suffer from disadvantages, such as high cost, difficult retrieval process, and incompatibility with enzymes and microorganisms (Saher et al., 2018; Keller et al., 2020; Wu et al., 2020), which significantly affect its large-scale industrial application on lignocellulosic biomass pretreatment.

##### Deep Eutectic Solvent Pretreatment Method

In order to solve the above-mentioned problems for the pretreatment process, deep eutectic solvents (DESs) were developed as a novel environmentally benign solvent with excellent characteristics of both organic solvents and ionic liquids (Smith et al., 2014). DESs are first proposed by Abbott as a versatile alternative to ionic liquids (Abbott et al., 2004), and are usually composed of a certain proportion of hydrogen bond acceptors (HBAs), such as quaternary ammonium salts and betaine, and hydrogen bond donors (HBDs), such as urea, multicomponent alcohol, and carboxylic acid (Abbott et al., 2001, 2004, 2007), which present properties, namely, low toxicity and cost, non-volatility, high thermal stability with excellent solubility, and environmental friendliness (Loow et al., 2017; Satlewal et al., 2018; Khan et al., 2021). The chemical properties of DESs are adjustable, so a targeted DES can be easily synthesized by selecting the appropriate HBD and HBA (Khan et al., 2021). Based on the above-mentioned characteristics, DESs can efficiently remove lignin (Francisco et al., 2012; Zhang et al., 2016a), dissolve most of hemicellulose (Xu et al., 2016; Yu et al., 2018), and, to a large extent, retain cellulose (Vigier et al., 2015; Ren et al., 2016), which is beneficial to the selective separation of the three major components of lignocellulosic biomass. Chen et al. adopted a DES solvent that is composed of 1:2 molar ratio of choline chloride and lactic acid (ChCl:LA) for the pretreatment of switch grass, corn stover, and *Miscanthus* under 800 W microwave radiation for 45 s, and found that the optimal removal rate of lignin, xylan, and dextran could be obtained at 80, 90, and 25%, respectively (Chen and Wan, 2018). Moreover, the purity of the recycled lignin could be 84–88% after enzymatic hydrolysis of the pretreated substrate. Wang et al. treated hybrid *Pennisetum* with ferric chloride and a DES prepared with choline chloride and glycerol at a reaction temperature of 120°C for 6 h, and the final lignin and hemicellulose removal rates could reach 78.88 and 93.63 wt%, respectively (Wang et al., 2020b). Notably, the retention rate of cellulose was 95.2% with purity of 80.94%, and the saccharification rate of cellulose was up to 99.5%. In addition, the reaction system has good recyclability that it can be reused for four times but the pretreatment performance after recycling is still considerable.

Despite having a lot of potential for the separation of lignocellulosic components, the high viscosity of DESs is an obvious disadvantage (Kumar and Sharma, 2017), and hemicellulose is underutilized (Penín et al., 2020).

##### Polar Aprotic Solvent Pretreatment

PASs include ketones, N,N-disubstituted amides, nitro hydrocarbons, nitriles, sulfoxides, sulfones, etc., and are generally accompanied with strong polarity, large dielectric constant, and weak alkaline and hydrogen bonding ability (Arnett and Douty, 1964; Reichardt, 1984). Normally, PASs have high boiling point, stable thermochemical properties, and strong dissolving power, and are miscible with water and most organic solvents. Therefore, they could dissolve lignin well and be easy to separate. The commonly used properties are shown in Table 2 (Kappe, 2004; Bak et al., 2018).

**Table 2:** Properties of usual polar aprotic solvents.

Properties	Sulfolane	DMSO	NMP	DMF	DMAc	THF	Dioxane	Acetone
Relative molecular mass	120.17	78.13	99.13	73.09	87.12	72.11	88.11	58.08
Density (g/cm^3^)	1.26	1.10	1.03	0.94	0.94	0.89	1.03	0.78
Boiling point (°C)	287.3	189.1	201.9	153.1	166.2	66	101.3	56.5
Freezing point (°C)	28.4	18.6	−24.4	−60.4	−20.1	−108	-	−94
Dielectric constant (ɛ_v_)	43.4^a^	46.7^b^	32.2^b^	36.7^b^	37.8^b^	7.58	2.51^b^	20.7
Dielectric loss (tanδ)	-	0.825	0.275	0.161	-	-	-	-
Dipole moment (debye)	4.69	3.96	4.09	3.8	3.72	1.75	0.45	2.85
Hildebrand parameters	27.2	26.6	23.6	24.1	23.3	-	-	-
Solvent polarity parameters	0.410	0.444	0.355	0.386	0.377	-	-	-
Viscosity (cP)	10.35^a^	2.0^b^	1.67^b^	0.9^c^	2.14^c^	0.55^c^	1.31^c^	0.32^b^
Flash point (°C)	177	89	86	58	63	−20	12	−20
Self-ignition point (°C)	528	302	270	445	445	321	180	465
Vapor pressure (kPa)	0.0091^a^	0.060^b^	0.050^b^	0.37^b^	0.13^b^	19.3^c^	5.33^b^	24.7^c^

a 30°C.

b 25°C.

c 20°C.

According to reports (Araque et al., 2008), a 50:50 (v/v) acetone: water solvent system was adopted for the pretreatment of *Pinus radiata* chips with sulfuric acid as catalyst at a temperature of 195°C and pH of 2 for 40 min. It was found that the removal rate of lignin and hemicellulose was 45.7 and close to 100%, respectively, accompanied with a dextran recycle rate of 46.3% and an enzymolysis rate of 71.8%. As another commonly used solvent, tetrahydrofuran (THF) could be prepared with 1,4-butanediol and furfural and proposed for pretreatment process (Hunter et al., 2006). Notably, after an 80/20 (w/w) THF/water mixed solution treated hardwood with 0.75 wt% H_2_SO_4_ as catalyst at 120°C for 1 h, the removal rates of lignin and xylan were around 50 and 70%, respectively, and the retention rate of cellulose was about 95% (Shuai et al., 2016). However, due to flammability and volatility, acetone and THF are rarely used for industrial pretreatment process.

Compared with acetone and THF, dimethyl sulfoxide (DMSO), N,N-dimethylformamide (DMF), and N,N-dimethylacetamide (DMAC) have higher boiling point, better solubility, stronger hygroscopicity, and higher skin permeability (Nishino et al., 2004; Wang et al., 2009; Tilstam, 2012). Li et al. pretreated wheat straw with an alkaline hydrogen peroxide solution containing 3% hydrogen peroxide twice (solid to liquid ratio of 1:25, g/ml, 70°C, 3 h). They then treated the residue (5 g) with lithium chloride/DMAC (LiCl/DMAC; 8%, w/w, 200 ml), stirred it at 110°C for 2.5 h, and then stirred it at room temperature for 12 h (Li et al., 2019). The removal rate of klason lignin was 95.02%, and the recovery rate of cellulose and xylan was 92.59 and 31.39%, respectively. Wei et al. pretreated corn stalk with 0.05 mol/L FeCl_3_-catalyzed DMSO at 120°C for 45 min (solid to liquid ratio of 1/10, kg/L; Wei et al., 2019). The results showed that the removal rate of lignin and xylan was 29.8 and 93.1%, respectively, and that the recovery rate of glucan was 91.9%. Chong et al. pretreated winter bamboo shoot shells by continuous extraction with 25 wt% ammonia water at 50°C for 24 h, followed by pretreatment with LiCl/DMF (6 wt% LiCl) at 50°C for 8 h (Chong et al., 2017). Notably, the removal rate of lignin and xylan could reach 68.8 and 42.4%, respectively, and the preservation rate of cellulose was 74.2%. However, due to flammability, toxicity, and violent reactivity with concentrated sulfuric acid, DMAC, DMSO, and DMF cannot be conducive to industrial application by itself. Moreover, in recent years, LiCl/DMAc, LiCl/DMSO, and LiCl/DMF have been mostly applied to dissolve cellulose (Wang et al., 2009, 2012; Hasani et al., 2013; Chong et al., 2017; Liu et al., 2019).

1,4-Dioxane has low boiling point (101°C) and strong dissolving power, and it can dissolve lignin well (Quesada-Medina et al., 2010; An et al., 2017). Zhang et al. used a new binary solvent system (methanol/dioxane) and p-toluenesulfonic acid (*p*-TsOH) as a catalyst to perform microwave-assisted pretreatment of poplar wood powder (Zhang et al., 2020c). The results showed that methanol/dioxane (75/25, V/V) exhibited the best performance with the solvent system for pretreatment, which could remove 88.3% lignin and 70.4% hemicellulose while retaining 83.1% cellulose. Enzymatic hydrolysis of poplar cellulose residue after pretreatment showed that the glucan conversion rate was close to the theoretical value (over 99%). In addition, the recycled lignin contained a small amount of condensed structure and narrow molecular weight distribution. However, the poor chemical stability performance of dioxane limits its application in industrial production.

On the other hand, some of the novel PASs, such as hexamethylphosphoramide (HMPA), isosorbide dimethyl ether (DMI), and methyl isobutyl ketone (MIBK), have also attracted the attention of researchers. HMPA and DMI have extremely high boiling point, excellent solubility and stability, large dipole moment, strong alkalinity, and low toxicity (Ding and Fang, 1993). However, due to toxicity, there are fewer applications of HMPA in the pretreatment of wood fiber biomass. Hou et al. employed a 3/1 ionic liquid BMIMOAC (1-butyl-3-methylimidazole acetate)/DMI solvent system to separate polysaccharides from wheat bran at 50°C for 3 h (Hou et al., 2015). The dissolution rate of wheat bran was 43.5%, accompanied with 71.8% extraction rate of polysaccharides. MIBK has a boiling point of 116.9°C, and is miscible with most organic solvents and slightly soluble in water. Katahira et al. used an MIBK/acetone/water system at 140°C and 0.1 M sulfuric acid as a catalyst to pretreat corn stover (Katahira et al., 2014). The results showed that the removal rate of lignin and hemicellulose was 18 and 44.7 wt%, respectively, and that the retention rate of cellulose was 64.4 wt%. However, MIBK is flammable, highly toxic, and irritating, which limits its application in industrialization. In addition, same with that of HMPA and DMI, the price of MIBK is relatively high and not conducive to industrial application.

## Green Solvent Pretreatment Method with Industrial Application Prospects

According to the concept of green chemistry from the overview of “Designing for a Green Chemistry Future in Science” by Julie B. Zimmerman and Paul T. Anastas et al., a green solvent should have the following characteristics: renewability, non-toxicity/low toxicity or low volatility, high thermal and chemical stability, degradability, recyclability, easy to separate and recycle, high efficiency, economical, not flammable and explosive, large scale use, low vapor pressure, and easy to store and transport (Anastas and Eghbali, 2010; Zimmerman et al., 2020). However, the above-mentioned solvents that are commonly used in the pretreatment process cannot be on par with these characteristics, and thus are limited in terms of industrial applications (Martín et al., 2007; Wang et al., 2016). Based on the above discussion, this review will introduce three pretreatment methods based on recyclable, non-toxic/low-toxic green solvents to achieve efficient selective separation and full component utilization of cellulose, hemicelluloses, and lignin, and provide an outlook of their industrial application prospects.

### Sulfolane Solvent

**Figure 3:**
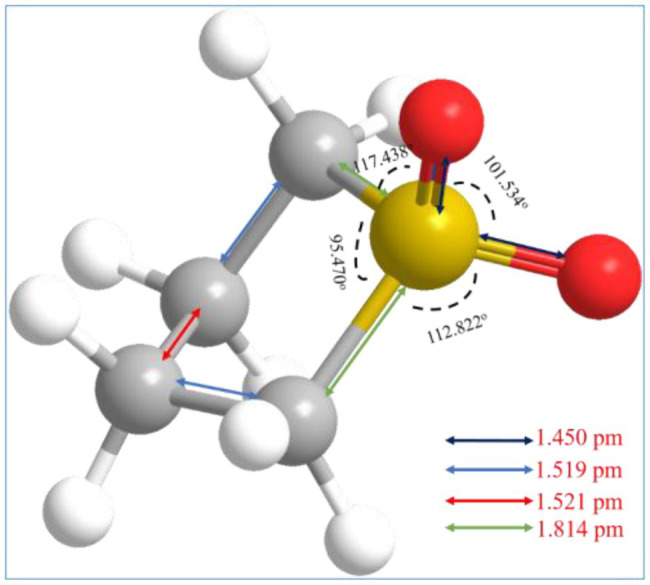
Schematic diagram of sulfolane.

Sulfolane is a cheap polar aprotic solvent with high boiling and flashing points, extremely low vapor pressure (1.93 kPa at 150°C), high thermochemical stability, and low level of volatility and toxicity, and it is originally used in the extraction of aromatic hydrocarbons in the petroleum industry (Singh et al., 2011a; Tilstam, 2012; Huang et al., 2014; Wang et al., 2016). Therefore, sulfolane could be defined as a green solvent according to the 12 principles. The basic structure diagram of sulfolane is shown in Figure 3. Because of the existence of sulfoxide double bond, it can be miscible with water and most organic solvents, etc. Notably, its special structure gives sulfolane the selective ability to extract aromatic hydrocarbons (Clark, 2000), and its outstanding compatibility with lignin instead of carbohydrates. Thus, sulfolane has a better industrial application prospect in delignification of lignocellulosic biomass (Tilstam, 2012).

Sulfolane is usually applied in the pretreatment of lignocellulosic biomass with the presence of water and catalysts. Previous studies by this research group have initially put forward that sulfolane is an effective and promising solvent for pretreatment of lignocellulosic biomass (Wang et al., 2016). When shrub willow was pretreated using a sulfolane solvent system with a ratio of substrate to solvent of 1:5 at 170°C for 1.5 h, the production rate of glucose and xylose could reach 78.2 and 56.6%, respectively. Notably, 84.7% of lignin removal rate could be obtained, accompanied with 13.9% of hemicellulose loss rate. Meanwhile, we investigated the effect of water and acid on the system and found that the anhydrous and acid-free conditions are conducive to the retention of cellulose. Finally, 80% of lignin removal rate could still be obtained after reusing sulfolane five times. Zhong et al. pretreated willow by alkaline catalysis with a sulfolane/water solvent system and prepared antioxidant lignin with high purity. The results showed that the lignin removal rate was 54% at 170°C with the sulfolane/water solvent system (mass ratio of 50/50; Zhong et al., 2020). Interestingly, when 4 wt% of NaOH was added as alkali catalyst, the lignin removal rate could gradually increase up to 94%, and the losses in glucose and xylose were controlled around 1.62 and 4.08%, respectively. The low molecular weight lignins collected by this pretreatment system had a phenolic hydroxyl group content (0.86–2.02 mmol/g), which was higher than that reported in the literature (0.2–0.45 mmol/g), which may be due to the breaking of aryl ether bonds or the phenolic hydroxyl group being more stable under relatively mild conditions. Furthermore, lignin components with high phenolic hydroxyl content had good free radical scavenging ability, indicating that they all had high antioxidant performance, which further increased the functionality of lignin. The research results showed that lignin extracted by sulfolane pretreatment has great potential to replace the expensive and relatively low efficiency antioxidants on the current market. Barahona et al. adopted sulfolane as an organic solvent for the pretreatment of bagasse to efficiently convert lignocellulosic biomass into hydrolyzed cellulose and high-purity lignin, and allow the recovery of valuable byproducts and improve the economic feasibility of the whole cellulosic ethanol process (Portero-Barahona et al., 2019). This research has shown that a total reductive sugar yield of 62.9% could be obtained with a 1:1 (v/v) water: sulfolane and 5% NaOH solution for 5 min at 140°C. The saccharification condition was to use 10 FPU cellulase/g for enzymatic hydrolysis of dry substrate at 50°C for 72 h, and the final glucose yield was 80.5%. Compared with the pretreatment of bagasse with water as medium, which also showed lower energy consumption, shorter reaction time, and higher glucose yield, the total reducing sugar and saccharinic acid production rate of sulfolane-TiO_2_ increased by 5 and 33%, respectively.

These studies indicated that sulfolane could selectively remove large amounts of lignin during the pretreatment process of lignocellulosic biomass. Furthermore, cellulose and hemicellulose can be preserved in large quantities by selecting the appropriate catalyst and dosage of water, which would also benefit the subsequent enzymatic hydrolysis saccharification process. Based on the physical and chemical properties and reported experimental studies, it is believed that sulfolane can be defined as a green solvent with low toxicity, high thermo-chemical stability, easy separation and recycling characteristics, high efficiency, low cost, and low steam pressure, etc. Therefore, sulfolane has broad application prospects in biomass pretreatment.

### γ-Valerolactone Solvent

**Figure 4:**
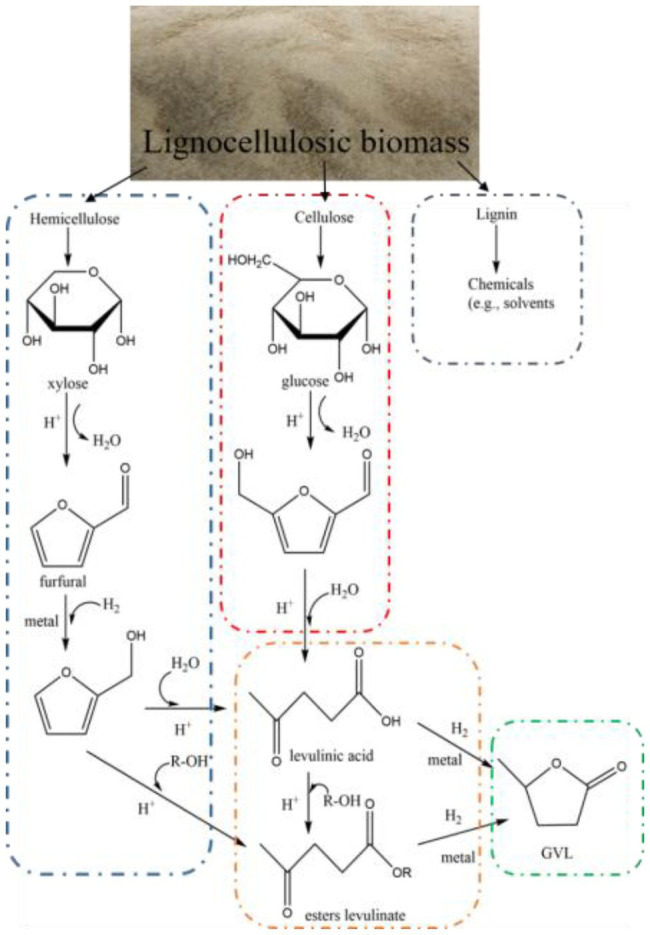
Synthesis path of GVL.

GVL is a well-known renewable organic compound (Yang et al., 2020), and it can be directly obtained from cellulose and lignocellulosic biomass (Alonso et al., 2013a,b; Cai et al., 2017). As shown in Figure 4, Alonso et al. introduced the general path for the preparation of GVL from lignocellulosic biomass by hydrogenation of levulinic acid. GVL is a thermo-chemical stable solvent with a low melting point of −31°C, high boiling point of 207°C, high flash point of 96°C and very low vapor pressure even at higher temperatures (0.65 kPa at 25°C; Alonso et al., 2013b). On the other hand, due to the following important characteristics, namely, easy and safe to store and transport in large quantities on a global scale, miscible with water, and contributor to biodegradation, GVL does not suffer from environmental problems and most risk factors, making it a safe material for large-scale use (Horváth et al., 2008). In addition, in the GVL/water system, it is easy to separate and recycle the GVL in pretreated waste liquid. By the combination of two-step lignin precipitation and vacuum distillation, more than 90% of lignin and GVL could be recycled from waste liquid (Lê et al., 2018).

It has been reported that a 80/20 (w/w) GVL/water composite system was used to pretreat hardwood with the presence of 0.75 wt% H_2_SO_4_ at 120°C for 1 h (Shuai et al., 2016). Up to 80% of the original lignin and 77% of the xylan could be selectively removed. Notably, more than 96–99% of the original cellulose can be retained. An enzyme loading of 15 FPU g^−1^ glucan was used to quantify the sugar conversion of the pretreated substrate, and the total glucose and xylose production rate was 99 and 100%, respectively. Moreover, by performing liquid carbon dioxide extraction on the pretreated slurry, more than 99% of sugars can be retained, accompanied with up to 99.5% recycle rate of GVL. This study found that most of the dissolved xylan was recycled in the form of oligomers in the pretreatment liquor, indicating that the hydrolysis of xylose and glycosidic bonds was very slow under these reaction conditions, which is benefit for reducing the degradation of sugar (Shuai et al., 2016). Wu et al. proposed a GVL/water system to combine *hybrid pennisetum* lignocellulosic biomass pretreatment with anaerobic digestion for biogas production and synthesis of lignin nanoparticles (LNPs; Wu et al., 2020). Notably, around 33% of lignin could be selectively removed without acid catalysis in the GVL/H_2_O = 50/50 (w/w) system at 150°C for 90 min, retaining about 92% of dextran and 85% of xylan sugar. Subsequently, the pretreated solid was used for anaerobic fermentation to produce methane. When the proportion of GVL in the solvent system reached 50%, the sample was hydrothermally pretreated to obtain the highest methane production (204 ml/g VS) because of the increase in solubility of lignin. Finally, a simple water sedimentation method was used to collect nearly 95% of LNPs from the pretreatment solution. After characterization, it was proved that LNPs can be prepared from the pretreatment waste of lignocellulosic biomass. Furthermore, the solvent in this system could be further recycled efficiently and proved that the GVL/H_2_O system is a sustainable green pretreatment method. Interestingly, GVL and the *p*-TsOH system were also adopted by Yang et al. for the pretreatment of hybrid poplar wood powders, and the optimal conditions were confirmed by characterizing the enzyme saccharification and lignin residues. The removal rates of lignin and hemicellulose reached 86.14 and 91.67%, respectively (Yang et al., 2020). Meanwhile, the enzymatic saccharification conversion rate of the cellulose residue could reach up to 84.84%. Finally, the related characterization of the separated lignin confirmed that the basic structure of lignin was generally maintained though a certain extent lignin was destroyed during the process. As a result, the lignin separated by this method has low average molecular weight, narrow molecular weight distribution, and high hydroxyl content, and can be easily used to produce fuels and chemicals, especially for lignin-phenolic resin synthesis (Pang et al., 2017).

In conclusion, GVL basically meets all the requirements of a green solvent, which could separate the three components efficiently and selectively. Though the cost of GVL is relatively high (53–80 dollars/kg), which partly limit its industrial application, the outstanding thermo-chemical stability endows GVL with excellent retrievability (Alonso et al., 2013b), especially in the production of value-added chemicals by direct liquefaction of lignocelluloses with the presence of a suitable heterogeneous catalyst (Liu et al., 2020). Thus, the GVL/H_2_O system pretreatment method has broad prospects in industrial applications.

### Dihydrolevoglucosenone (Cyrene) Solvent

**Figure 5:**
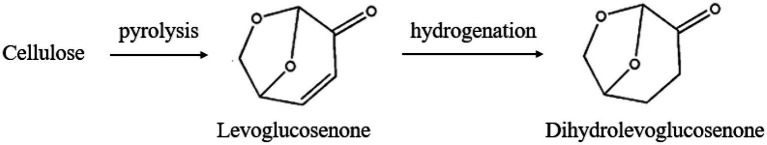
Synthesis path of dihydrolevoglucosenone.

Cyrene is a promising novel bio-based solvent derived from cellulose through two simple paths, which are shown in Figure 5 (Zhang et al., 2016b). Just like GVL, Cyrene has characteristics, such as regeneration and economic feasibility and high boiling point (about 203°C). In addition, Table 3 shows the similarities in the physical properties of N-methyl pyrrolidone (NMP), DMF, sulfolane, and Cyrene (Sherwood et al., 2014; Zhang et al., 2016b). Moreover, studies also shown that Cyrene has no mutagenic properties and is relatively non-toxic, which is one of the green organic solvents that can replace toxic polar solvents (Sherwood et al., 2014; Zhang et al., 2016b; Camp, 2018). In the pharmaceutical industry (Camp et al., 2020), synthetic chemistry (Zhang et al., 2016b; Bousfield et al., 2019), materials chemistry and other fields (Marino et al., 2019; Poon and Zhitomirsky, 2020), Cyrene has already been used as a sustainable solvent. Previous studies have shown that Cyrene has good lignin solubility due to its relatively high HBA capacity (0.61) based on the Kamlet–Abboud–Taft parameter. Furthermore, it is also highly miscible with water without forming an azeotrope, so it is possible to recycle it from its mixture with water by simple distillation (Camp, 2018; Meng et al., 2020b).

**Table 3:** Physical properties of some polar aprotic solvents.

	Cyrene	NMP	DMSO	DMF	DMAc	Sulfolane
Dipolarity	0.93	0.90	1.00	0.88	0.85	0.96
δ_D_/MPa^0.5^	18.8^a^	18.0	18.4	17.4	16.8	20.3
δ_P_/MPa^0.5^	10.6^a^	12.3	16.4	13.7	11.5	18.2
δ_H_/MPa^0.5^	6.9^a^	7.2	10.2	11.3	10.2	10.9
MP/°C	﹤-18	−24	18.6	−60.5	−20.1	28.4
BP/°C	203	202	189	153	165	282
ρ/g cm^−1^	1.25	1.03	1.10	0.94	0.94	1.26
V_m_/cm^3^mol^−1^	102.5	96.5	-	77.0	-	-

a Calculated with HSPiP software.

The research group of Meng treated poplar in a Cyrene/water solution with a solvent ratio of 4:1 at 120°C for 60 min, and found that the strong hydrogen bond that formed between Cyrene and water is beneficial for the cleavage of lignin–carbohydrate linkages (Meng et al., 2020b). Therefore, the Cyrene/water ratio played a key role in the solubility of lignin. In addition, they also found that the pretreatment process could be conducted under mild conditions to reduce lignin condensation and β-O-4 bond breakage without affecting the removal and dissolution of lignin during the pretreatment of *Populus trichocarpa x deltoids* with the Cyrene/water system. Interestingly, some unwanted inhibitors formed during longer pretreatment time because of the high concentration of Cyrene in the cosolvent mixture. Subsequent studies have found that dilute alkali incubation was a good method to overcome the negative effects of high Cyrene concentration or long pretreatment time. In order to better understand how Cyrene works during the pretreatment of biomass, the researchers also tested the degree of polymerization of cellulose and lignin. Lignin integrity is well preserved, and the recovery rate of original lignin is over 60%. Moreover, all the Cyrene lignin has high contents of syringyl units and β-O-4 linkages. At present, however, a large amount of water must be distilled to recover pure Cyrene. Therefore, how to effectively recycle Cyrene from the cosolvent system and reuse it efficiently still needs further study.

Combined with the current research, although Cyrene is currently relatively expensive, and the cost of water used during the retrieval of Cyrene is comparatively high (Clarke et al., 2018; Camp et al., 2020), its outstanding performance on selective degradation of lignin cannot be ignored. As a biodegradable and bio-based green solvent, Cyrene has a great prospect in the pretreatment of lignocellulosic biomass (such as grass, softwood, and agricultural energy crops), and the byproduct of Cyrene lignin can be used as a high value-added product in aromatic compounds, carbon fiber, polyurethane precursors, etc (Meng et al., 2020b).

## Conclusion

Based on the discussion of traditional pretreatment methods, including biological, physical, chemical and combined processes, this study focuses on the discussion of pretreatment process with recyclable and non-toxic/low-toxic green solvents, such as PASs, ionic liquids, and deep eutectic solvents. According to the classical definition of green solvent, PASs are believed to be one of the most promising green solvent systems with extremely high thermo-chemical stability, low skin permeability, and low saturated vapor pressure, and they can selectively remove lignin and refrain from unwanted loss of hemicellulose and cellulose. Notably, bio-derived PASs, such as GVL and Cyrene, exhibited excellent properties, namely, renewability, degradability, low toxicity, easy recovery, high efficiency, and stability. Moreover, their advances in biotechnology, synthetic chemistry, and chemical engineering, which are in line with the concept of sustainable development, are leading to a new concept of “closed-loop” biorefinery when using biomass-derived solvents to convert biomass into liquid fuels and valuable products (Meng et al., 2020b),. Briefly, this minor review proposed considerable pretreatment methods with green solvents, indicating a promising application prospect for industrial bio-refinery process.

## Author Contributions

XY: writing and data curation. LW: methodology. XP: checking and review. CL: writing and editing. JJ: supervision. KW: conceptualization and supervision. All authors contributed to the article and approved the submitted version.

### Conflict of interest

The authors declare that the research was conducted in the absence of any commercial or financial relationships that could be construed as a potential conflict of interest.

The handling editor declared a past co-authorship with several of the authors (JJ and KW).
